# Automatic classification of the vertebral endplate lesions in magnetic resonance imaging by deep learning model

**DOI:** 10.3389/fsurg.2023.1172313

**Published:** 2023-06-22

**Authors:** Tito Bassani, Andrea Cina, Fabio Galbusera, Luca Maria Sconfienza, Domenico Albano, Federica Barcellona, Alessandra Colombini, Andrea Luca, Marco Brayda-Bruno

**Affiliations:** ^1^ IRCCS Istituto Ortopedico Galeazzi, Milan, Italy; ^2^ Spine Center, Schulthess Clinic, Zurich, Switzerland; ^3^ Department of Health Sciences and Technologies, ETH Zurich, Zurich, Switzerland; ^4^ Dipartimento di Scienze Biomediche per la Salute, Università Degli Studi di Milano, Milan, Italy; ^5^ Complex Unit of Radiology, Department of Diagnostic and Interventional Radiology, Azienda Socio Sanitaria Territoriale (ASST) Lodi, Lodi, Italy

**Keywords:** spine, endplate lesions, osteochondrosis, artificial intelligence, deep learning, automatic classification, convolutional neural networks

## Abstract

**Introduction:**

A novel classification scheme for endplate lesions, based on T2-weighted images from magnetic resonance imaging (MRI) scan, has been recently introduced and validated. The scheme categorizes intervertebral spaces as “normal,” “wavy/irregular,” “notched,” and “Schmorl's node.” These lesions have been associated with spinal pathologies, including disc degeneration and low back pain. The exploitation of an automatic tool for the detection of the lesions would facilitate clinical practice by reducing the workload and the diagnosis time. The present work exploits a deep learning application based on convolutional neural networks to automatically classify the type of lesion.

**Methods:**

T2-weighted MRI scans of the sagittal lumbosacral spine of consecutive patients were retrospectively collected. The middle slice of each scan was manually processed to identify the intervertebral spaces from L1L2 to L5S1, and the corresponding lesion type was labeled. A total of 1,559 gradable discs were obtained, with the following types of distribution: “normal” (567 discs), “wavy/irregular” (485), “notched” (362), and “Schmorl's node” (145). The dataset was divided randomly into a training set and a validation set while preserving the original distribution of lesion types in each set. A pretrained network for image classification was utilized, and fine-tuning was performed using the training set. The retrained net was then applied to the validation set to evaluate the overall accuracy and accuracy for each specific lesion type.

**Results:**

The overall rate of accuracy was found equal to 88%. The accuracy for the specific lesion type was found as follows: 91% (normal), 82% (wavy/irregular), 93% (notched), and 83% (Schmorl's node).

**Discussion:**

The results indicate that the deep learning approach achieved high accuracy for both overall classification and individual lesion types. In clinical applications, this implementation could be employed as part of an automatic detection tool for pathological conditions characterized by the presence of endplate lesions, such as spinal osteochondrosis.

## 1. Introduction

**Figure 1:**
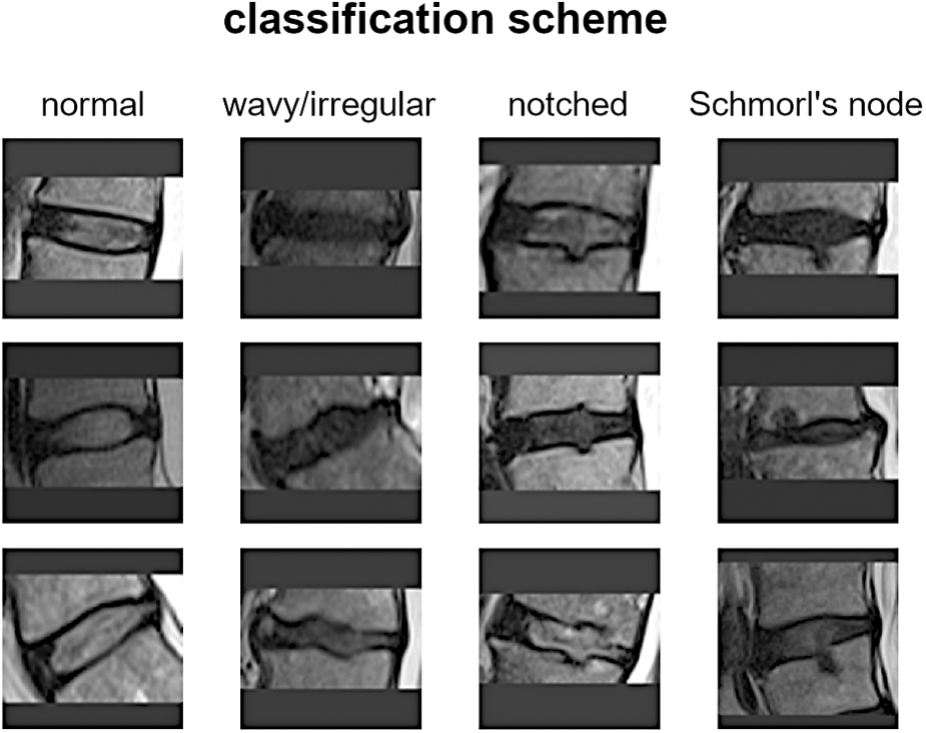
Classification scheme with examples for the endplate lesion types.

The vertebral endplate is a crucial structure situated between the vertebral body and the intervertebral disc. It serves as a mechanical interface between the rigid bone and the flexible disc, playing a vital role in maintaining the morphological integrity and physiological function of the disc. Traditionally, most endplate pathologies or lesions were classified as Schmorl's nodes, which refer to the protrusion of disc tissue through the endplate into the vertebral marrow (1). Schmorl's nodes are often asymptomatic and are frequently discovered incidentally during clinical practice through magnetic resonance imaging (MRI) scans. However, in recent years, several studies have aimed to differentiate Schmorl's nodes from other morphological abnormalities such as indentations or defects on the endplate (2). For instance, Feng et al. distinguished among focal, corners, and erosive endplate lesions (3). Another study employed a more comprehensive endplate scoring system based on T1-weighted MRI scans to correlate lesion presence with the transport of a contrast agent into the intervertebral disc (4). More recently, Brayda-Bruno et al. proposed a novel classification scheme for endplate lesions based on T2-weighted MRI scans, which simplified and adapted the aforementioned scoring system (5). In this scheme, the intervertebral spaces are classified as “normal,” “wavy/irregular,” “notched,” and “Schmorl's node” (Figure 1). This classification scheme has been validated in terms of reliability between different observers and within the same observer, and has been evaluated for potential associations with disc degeneration, disc herniation, and low back pain (5–7).

The presence of endplate lesions and their relation to spinal pathologies, such as disc degeneration, disc herniation, and juvenile vertebral osteochondrosis (Scheuermann's disease), has been extensively investigated and confirmed in numerous studies (3, 8–17). The development of an automatic tool for classifying the type of endplate lesion would offer advantages in clinical practice by reducing the workload for operators and the time required for diagnosis. In addition, such a tool would enable the rapid and accurate processing of large datasets for research purposes. In this regard, artificial intelligence techniques have been increasingly employed in spine research to predict parameters from radiological images, aiming to provide reproducible and reliable evaluations (18, 19). Deep learning methods utilizing convolutional neural networks have been applied to recognize vertebral landmarks, reconstruct spinal alignment from radiographic images (20–23), and automatically segment and identify vertebrae in computed tomography and MRI scans (24). Moreover, specific applications, like the “SpineNet” computer vision-based system, have been developed to extract relevant measurements, such as Pfirrmann grade, Modic changes, spinal stenosis, and disc herniation, from MRI scans (23, 25–28). As a novel contribution, the present work exploits a deep learning model based on convolutional neural networks to automatically classify the type of endplate lesion according to the scheme proposed by Brayda-Bruno et al. (5), in a dataset of 1,559 intervertebral spaces obtained from retrospectively collected subjects.

## 2. Materials and methods

### 2.1. Classification of the endplate lesions

The scoring system for classifying intervertebral spaces based on endplate lesions, as described in reference (5), is summarized as follows: (i) “normal” classification is assigned when no lesions are visually identified in the sagittal MRI slices that encompass the intervertebral space. The endplates appear structurally intact without any noticeable abnormalities; (ii) “wavy/irregular” classification is given when there are no specific lesions detectable in the intervertebral space, but the shape of at least one of the endplates exhibits alterations compared with the typical curvature seen in a healthy intervertebral space. The endplate may appear wavy or irregular in shape; (iii) “notched” classification is assigned if a small lesion is visible in at least one slice of the sagittal MRI. The lesion has a V-shaped or circular appearance and is present on one or both of the endplates. These notches may indicate small defects or indentations on the endplates; (iv) “Schmorl's node” classification is used when a deep focal defect is observed in the vertebral endplate. The lesion has a smooth margin and a rounded appearance. Schmorl's nodes are characterized by disc tissue protruding through the endplate and into the vertebral marrow.

It is important to note that the additional class “fracture” mentioned in the reference study was excluded in the present work due to its limited representation in the considered group of subjects. Therefore, the focus of the study was on classifying intervertebral spaces into normal, wavy/irregular, notched, and Schmorl's node categories based on the identified lesions in the MRI scans (Figure 1). For more detailed information on the scoring system and its validation, please refer to the original reference (5).

### 2.2. Dataset and image processing

**Figure 2:**
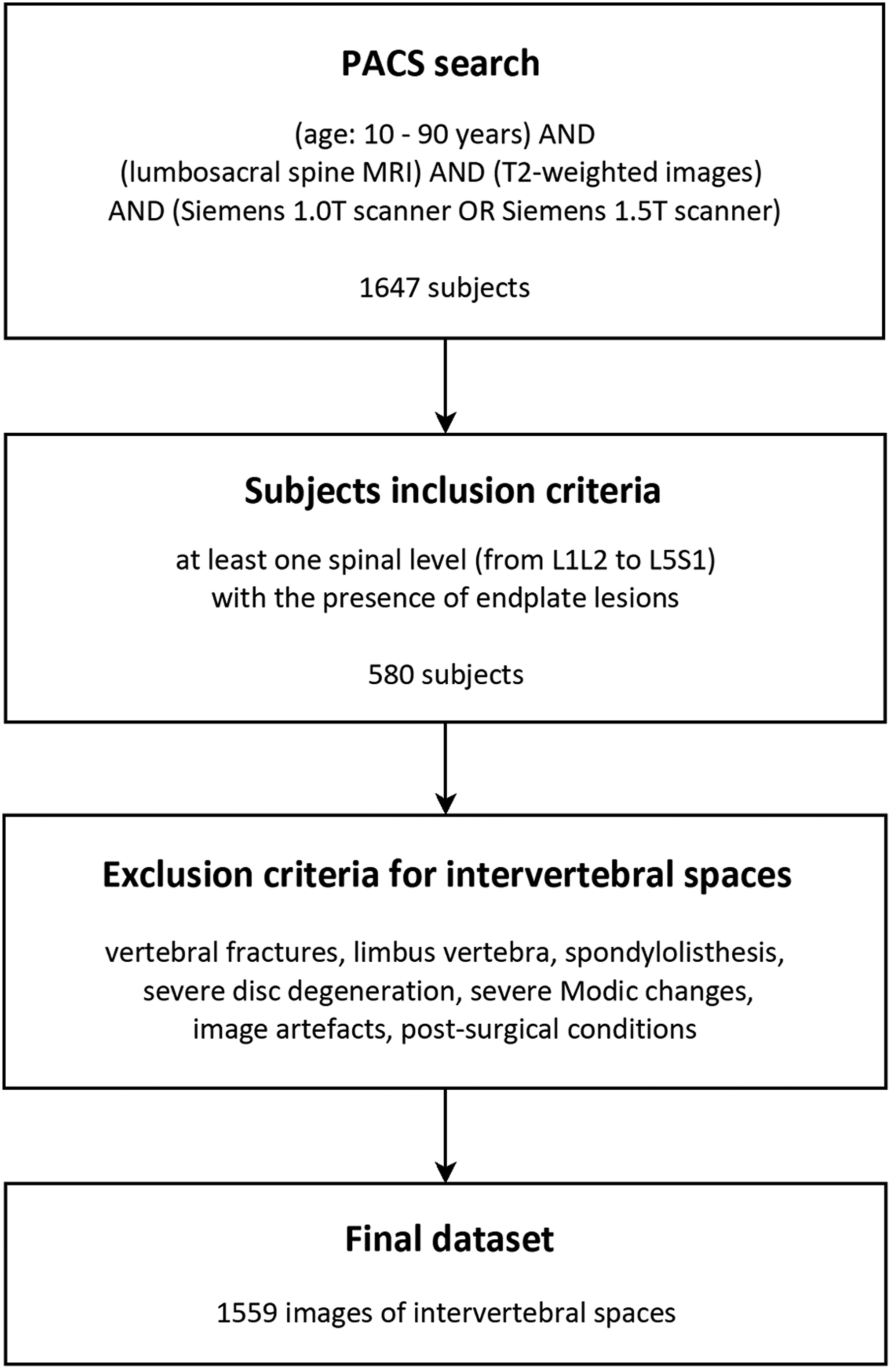
Chart diagram illustrating the workflow for the image selection.

In this study conducted at the IRCCS Istituto Ortopedico Galeazzi in Milan, Italy, a retrospective search was performed using the Picture Archiving and Communication System (PACS) to identify anonymized subjects who had undergone lumbosacral MRI scans between June 2016 and January 2018 (Figure 2). The search included a Caucasian population ranging in age from 10 to 90 years. The MRI scans were performed using either 1.5 T scanners (Avanto and Espree, Siemens AG, Erlangen, Germany) or a 1.0 T scanner (Harmony, Siemens AG, Erlangen, Germany). The resulting dataset partially integrates that exploited in our previous study by Brayda-Bruno et al. (5). T2-weighted sagittal MRI scans of the lumbosacral spine were collected for consecutive patients. Only subjects with at least one spinal level showing the presence of endplate lesions were included in the study. An experienced radiologist reviewed the MRI scans and identified the lesion type for each intervertebral space from levels L1L2 to L5S1, according to the classification scheme proposed by Brayda-Bruno et al. (Figure 1).

**Figure 3:**
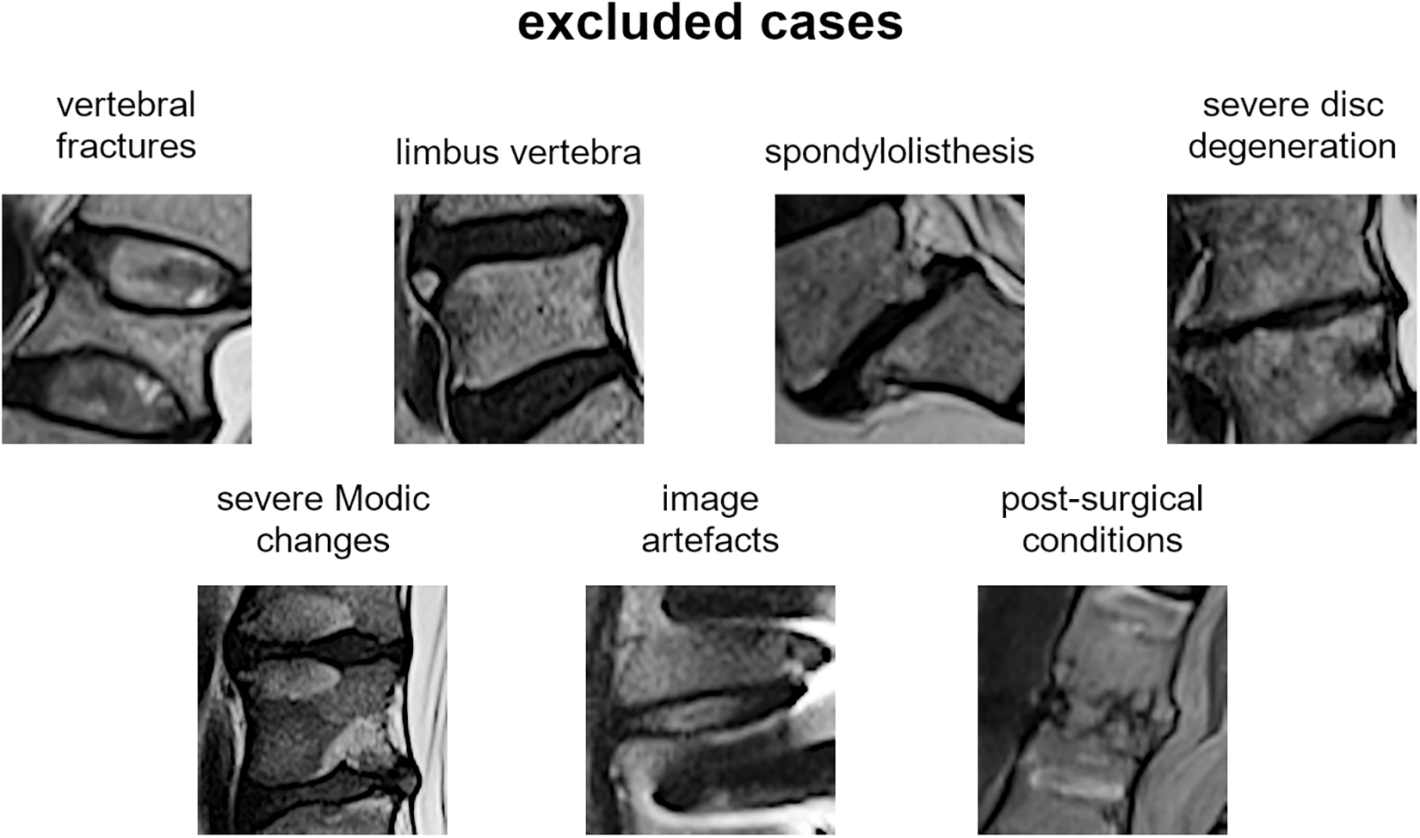
Excluded cases in the selection of the intervertebral spaces.

**Figure 4:**
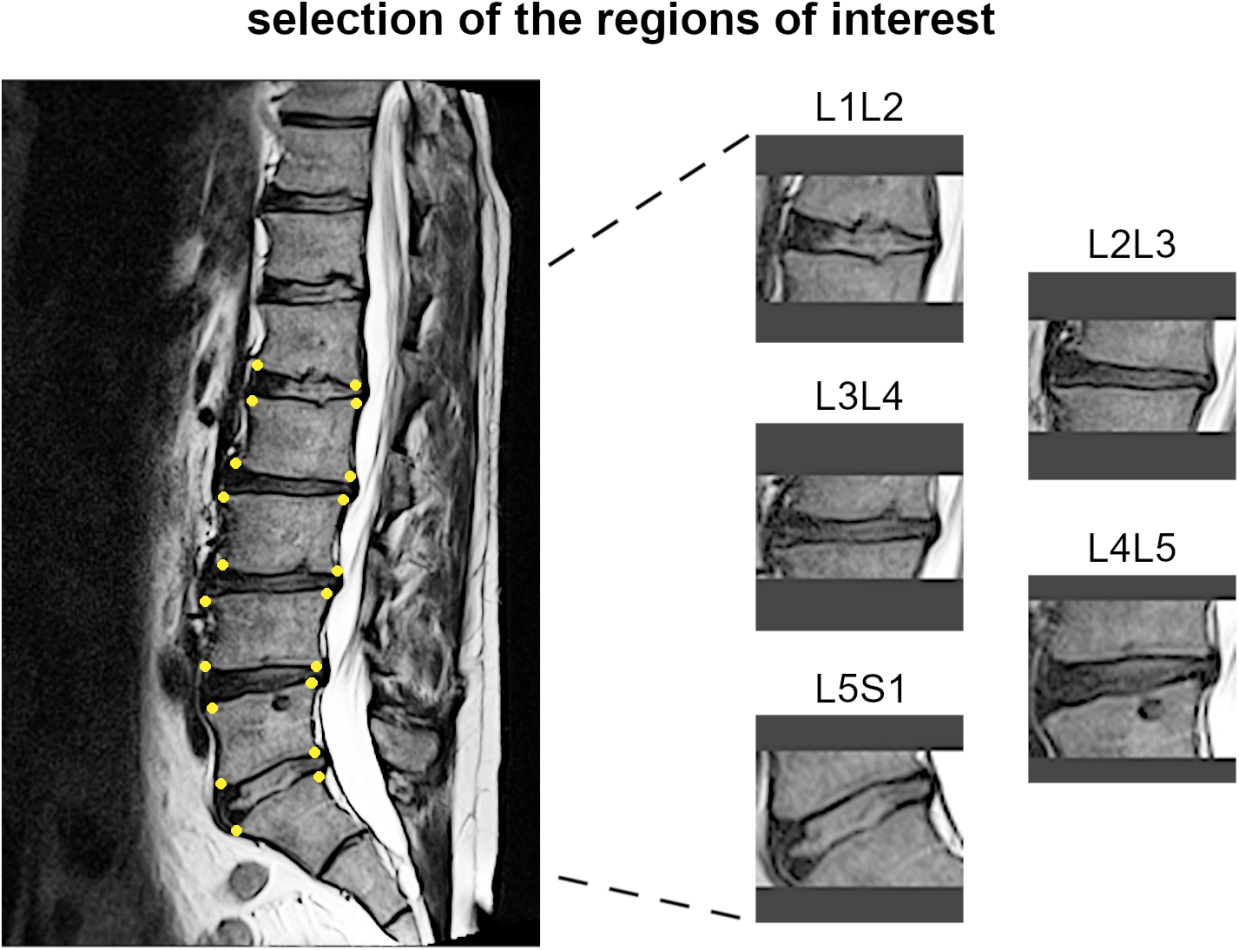
Example of identification of the region of interests for one subject, with the manually handpicked vertebral corners of the upper and lower endplates depicted as yellow dots (left panel) and the obtained images for the corresponding spinal levels (right panel).

The most representative slice image, typically the middle slice, was selected for each patient. Spinal levels with certain conditions, such as vertebral fractures, limbus vertebra, spondylolisthesis, severe disc degeneration [graded Pfirrmann 5 (29)], severe Modic changes (30) (extending over 25% or more of the vertebral height), as well as image artifacts and postsurgical conditions (e.g., interbody fusion), were excluded from the analysis (Figure 3). The selected slice images were imported into a custom tool developed using MATLAB software (MathWorks Inc., Natick, MA, USA). The tool allowed the manual identification of the region of interest (ROI) for the intervertebral spaces by selecting the corners of the corresponding upper and lower vertebral endplates (Figure 4, left panel). The images were preprocessed by contrast enhancement (saturation of the bottom 1% and the top 1% of all pixel values), and the ROIs encapsulating the disc and approximately 30% of the upper and lower vertebrae, along with some spinal canal cerebrospinal fluid, were resized to 128 × 128 pixels. Gray padding was applied to fill the image height (Figure 4, right panel). A total of 1,559 images of intervertebral spaces were obtained, distributed among the following lesion types: “normal” (567 discs), “wavy/irregular” (485), “notched” (362), and “Schmorl's node” (145).

The dataset was divided randomly into a training set (1,247 discs, 80%) and a validation set (312 discs, 20%), while maintaining the original distribution of the four lesion types in each set. A pretrained deep learning model based on the ResNet18 architecture, originally trained on the ImageNet dataset, was used for image classification. The model was fine-tuned using the training set by progressively unfreezing the weights of the deeper layers in multiple steps with increasing numbers of epochs. The network was trained for 100 epochs, with a learning rate of 0.001 that was reduced by a factor of 10 if the loss did not improve for 10 epochs. To address the class imbalance issue in the dataset, sample weighting correction was applied during the training process (31, 32). This correction involved assigning different weights to individual samples, which influenced their probability of being selected during the sampling process. The goal was to give more relevance to the underrepresented classes, ensuring that they had a higher chance of being chosen during training. This approach helps to balance the training process and prevent the model from favoring the majority class. A weighted random sampler function was used with a batch size of 32. This allowed for resampling the images from the underrepresented classes after each batch set was preprocessed using augmentation techniques such as small shifts, rotations, and flips, in order to increase the variety of the dataset and the robustness of the model.

The validation set was used to evaluate the performance of the trained model. The overall accuracy was calculated by dividing the number of correct predictions by the total number of samples in the validation set. In addition, the accuracy level for each specific lesion type, referred to as class sensitivity, was determined by dividing the number of correct predictions for that type by the number of samples in that specific class. Model calibration was performed using the Platt scaling method (33). This approach rescaled the predicted probabilities of the model to make them more representative of the true likelihood of occurrences of the classes present in the training data. It helped to mitigate potential overconfidence of the model, especially in the presence of unbalanced classes (34). To provide accurate estimations of the performance metrics, bootstrap resampling was carried out using a 1-vs.-all approach. A total of 500 iterations were run on the validation set to calculate the mean values of metrics such as area under the curve (AUC), accuracy, precision, recall, specificity, and F1-score. In addition, 95% confidence intervals were computed for these metrics to provide a measure of their variability and reliability. The image resizing and the implementation of the deep learning model were performed using Python, utilizing the open-cv library for image processing and the PyTorch framework (35) for the deep learning model. These tools and libraries are commonly used in computer vision and deep learning applications.

## 3. Results

**Figure 5:**
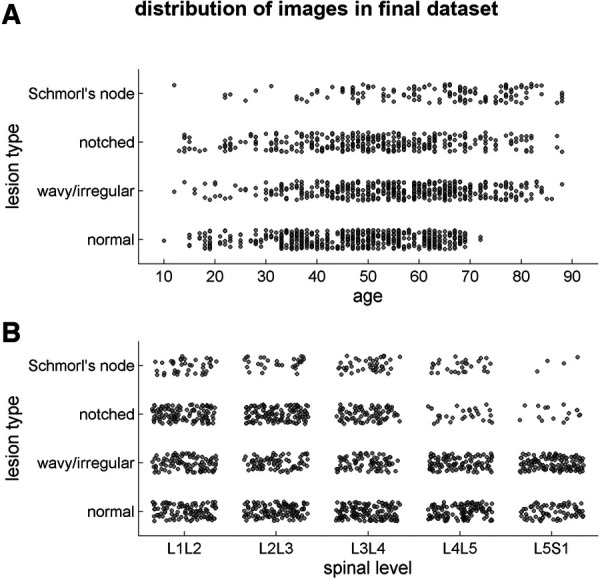
Scatter plots illustrating the distribution of the lesion types in the final image dataset (1,559 samples) depending on the age of the subjects (**A**) and on the spinal level (**B**).

The final dataset included subjects with an age range of 10–88 years, with a mean age of 52 ± 15 years. The gender distribution was equal, with 50% males and 50% females. The presence of endplate lesions generally increased with age (Table 1 and Figure 5A). The distribution of lesion types across age groups showed that normal cases were more common in subjects younger than 70 years, while Schmorl's node cases were predominantly found in subjects older than 40 years. The distribution of lesion types along the spinal levels was similar for normal and wavy/irregular types. Notched endplates were more prevalent at higher lumbar levels, and Schmorl's node cases were less represented only at the L5S1 level (Table 2 and Figure 5B).

**Table 1:** Distribution of the endplate lesion types in the age range (indicated as the number of cases and corresponding percentage in the considered age).

	Normal, *n* (%)	Wavy/irregular, *n* (%)	Notched, *n* (%)	Schmorl's node, *n* (%)
10–20	25 (51)	10 (20)	13 (27)	1 (2)
21–30	32 (42)	13 (17)	26 (34)	5 (7)
31–40	124 (53)	50 (22)	51 (22)	8 (3)
41–50	134 (41)	90 (27)	86 (26)	21 (6)
51–60	130 (37)	118 (33)	83 (24)	22 (6)
61–70	119 (34)	131 (37)	63 (18)	37 (11)
71–80	2 (2)	57 (45)	29 (23)	37 (30)
81–90	0 (0)	16 (39)	11 (27)	14 (34)

**Table 2:** Distribution of the endplate lesion types in the spinal levels (indicated as the number of cases and corresponding percentage in the considered level).

	Normal, *n* (%)	Wavy/irregular, *n* (%)	Notched, *n* (%)	Schmorl's node, *n* (%)
L1L2	105 (30)	97 (28)	109 (31)	40 (11)
L2L3	123 (34)	79 (22)	126 (35)	30 (8)
L3L4	137 (40)	79 (23)	83 (24)	43 (13)
L4L5	127 (44)	106 (37)	28 (10)	28 (10)
L5S1	75 (34)	124 (56)	16 (7)	4 (2)

**Figure 6:**
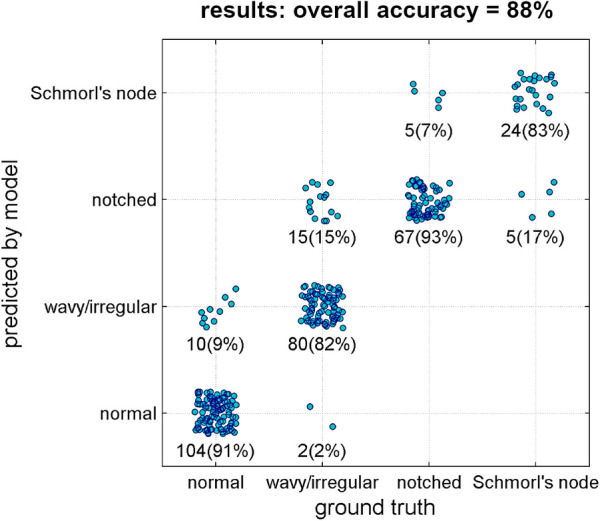
Results of the classification model (ground truth type vs. predicted type). Each point represents one single validation sample. Number of samples and corresponding percentage in the considered ground truth type are reported as well.

The deep learning model achieved an overall accuracy of 88% when processing the validation set (Figure 6). Out of 312 samples, 275 were correctly classified by the model. The accuracy rates for the specific lesion type were found as follows: 91% for normal type (104 in 114 samples), 82% for wavy/irregular (80 in 97), 93% for notched (67 in 72), and 83% for Schmorl's node (24 in 29). The model performance metrics, calculated using bootstrap resampling, showed average values of 96% for AUC, 88% for accuracy, 87% for precision, recall, and F1-score, and 96% for specificity (Table 3).

**Table 3:** Model performance metrics (% values), indicated as mean and 95% confidence interval.

	AUC	Accuracy	Precision	Recall	Specificity	F1-score
Model performance	96 (94–98)	88 (84–91)	87 (82–91)	87 (83–91)	96 (94–97)	87 (82–91)

## 4. Discussion

The presence of endplate lesions in the lumbosacral spine was found in 35% of the subjects included in the study (580 out of 1,647 cases, Figure 2), which is consistent with the prevalence observed in a previous study (37%) using a similar methodology in a dataset of Caucasian subjects from the same institute but with a narrower time period (5). However, studies involving Chinese populations and random selection of subjects, including both back pain patients and healthy volunteers, have reported higher prevalence rates of approximately 60% (7, 12, 36). These variations in prevalence could be attributed to differences in assessment methodologies, subject inclusion criteria, and the presence of other spinal pathologies. Unfortunately, other studies assessing Caucasian populations did not provide the prevalence inside subjects but only in terms of evaluated discs, thus preventing a direct and consistent comparison with the prevalence findings of the current study (9, 37).

The final dataset obtained after applying exclusion criteria for intervertebral spaces (Figure 3) showed a similar distribution of lesion types across age ranges and spinal levels (Figure 5 and Tables 1, 2). However, there was a tendency for higher lesion prevalence with increasing age, which is in line with previous studies (3, 11). Schmorl's node was the least prevalent type (145 out of 1,559 discs), while wavy/irregular and notched types were similarly represented (485 and 362, respectively). In this regard, the sample weighting correction procedure used in training the deep learning model helped mitigate the effects of imbalance among the lesion types by adjusting their proportional contribution.

The validation of the model demonstrated a strong overall accuracy rate (88%, Figure 6), with similarly high percentages of correct predictions for each lesion type (ranging from 82% to 93%). The model performance metrics, including accuracy, precision, recall, specificity, and F1-score, ranged from 87% to 96% (Table 3), indicating the model's reliability and potential utility for research and clinical purposes. Specifically, the implementation of the model could facilitate the efficient processing of large image datasets in descriptive studies reducing the workload for operators, and potentially be integrated into existing systems for automatic detection of pathological conditions characterized by endplate lesions such as spinal osteochondrosis. In this regard, the potential integration with existing systems such as “SpineNet” (23, 25–28) is a valuable consideration. “SpineNet” is indeed currently capable of automatically recognizing the presence of endplate lesions, although as a binary outcome (presence or absence). In contrast, the present model developed in the study focuses on classifying the specific types of endplate lesions. By integrating the two approaches, it would be possible to enhance the capabilities of the system and provide more comprehensive information.

The present study has the following limitations. The number of images in the dataset is moderate for a deep learning approach, and the imbalance among the lesion types, particularly for Schmorl's node, poses a challenge. Although the trained model demonstrated high accuracy levels for each lesion type, larger and more balanced datasets, along with cross-validation analyses, would be beneficial for further model refinement. Another limitation is the arrangement of the training and validation sets, which were randomly selected at the image level rather than the subject level. The final dataset was indeed characterized by 1,559 images from 580 subjects, implying a variable number of spinal levels from each single subject. Such an approach was required to guarantee the fundamental aspect of preserving in each subset the original distribution of the four lesion types. This could result in data from the same individual being present in both subsets, although this should not significantly affect the results. Lastly, with regard to the evaluated subjects, no clinical data or relevant information regarding comorbidities or specific reasons for undergoing MRI scans could be retrieved from the retrospective search because they were not stored in the PACS. Although this information would be useful in characterizing the evaluated population and understanding the context of the observed endplate lesions, its absence does not directly impact the classification of lesion types by the developed model.

In conclusion, although further validation and refinement are necessary, the deep learning model demonstrated promising performance levels for the automatic detection and classification of endplate lesions. The model has potential applications in both research and clinical settings, but larger datasets and external validation (exploiting data from other populations, study groups, and scanning devices) are needed to establish its robustness and generalizability.

## Data Availability

The raw data supporting the conclusions of this article will be made available by the authors without undue reservation.
